# Clinical Investigation into Posterior Capsule Rupture in Phacoemulsification Operations Performed by Surgery Trainees

**DOI:** 10.1155/2020/1317249

**Published:** 2020-02-12

**Authors:** Haiqing Bai, Lin Yao, Haitao Wang

**Affiliations:** ^1^Department of Ophthalmology, The Affiliated Hospital of Qingdao University, Qingdao, China; ^2^Qingdao Xinshijie Eye Hospital, Qingdao, China

## Abstract

This retrospective cohort study investigated the occurrence rate, correlative factors, and prognosis of posterior capsule rupture (PCR) in phacoemulsification operations conducted by surgery trainees. This study assessed the first 200 phacoemulsification surgeries performed by six surgery trainees between August 2016 and December 2018. Cases were divided into two groups depending on whether they fell within the first 100 surgeries performed by a trainee (first 100 cases group) or the last 100 surgeries (last 100 cases group). The following clinical data were analyzed: the occurrence rate of PCR, whether this complication arose in the phaco or irrigation/aspiration (IA) phase, the occurrence of vitreous loss, retinal detachment, and dropped nucleus, the site of intraocular lens (IOL) implantation, and postoperative visual acuity. Thirty-nine of the 1200 cases (3.25%) experienced PCR. The occurrence rates of PCR and vitreous loss were higher in the first 100 cases group than in the last 100 cases group (*P*=0.015 and *P*=0.017). PCR occurred more frequently in the phaco phase in the first 100 cases group and in the IA phase in the last 100 cases group (*P*=0.012). There was no difference between the two groups in terms of site of IOL implantation, the occurrence of retinal detachment or dropped nucleus, and postoperative visual acuity. With a supervising surgeon and the selection of suitable cases, the occurrence rate of PCR in phacoemulsification operations performed by surgery trainees could be controlled to the desired level. The phase in which PCR most frequently occurred and the likelihood of vitreous loss differed depending on the level of surgical experience of the trainees. It is a lengthy process for surgery trainees to reach the stage at which they can manage PCR and complex cataract surgery independently.

## 1. Introduction

Posterior capsule rupture (PCR) is a common and severe complication of phacoemulsification [1]. A ruptured posterior capsule makes the operation more complicated, especially when vitreous prolapse occurs. PCR can also result in the postoperative impairment of visual acuity. Moreover, without appropriate treatment, PCR can cause many additional complications such as retinal detachment, macular edema, uveitis, glaucoma, and intraocular lens (IOL) dislocation [2]. Previous studies have found the occurrence rate of PCR in phacoemulsification surgery to be between 1.9 and 5.2%, and to be lower when surgeons have greater experience with the technique [3–7]. It would therefore be expected that phacoemulsification conducted by surgery trainees should be associated with a much higher occurrence rate of PCR.

At present, there are relatively few published studies that examine the occurrence of PCR in phacoemulsification operations performed by surgery trainees. Furthermore, the occurrence rate of PCR in surgeries conducted by trainees with different levels of surgical experience remains unclear. In this study, we enrolled six surgery trainees and collected data regarding their first 200 phacoemulsification cases. The purpose of this study was to analyze the occurrence rate, correlative factors, and prognosis of PCR in phacoemulsification surgeries conducted by trainees with different levels of surgical experience.

## 2. Materials and Methods

### 2.1. Subjects

This retrospective cohort study examines the 39 cases of PCR (15 males and 24 females) observed in the first 200 phacoemulsification surgeries conducted by six surgery trainees between 1 August 2016 and 31 December 2018. All of the surgery trainees had at least five years' experience in microsurgery, mastered incision, capsulorhexis, irrigation/aspiration (IA), and IOL implantation. The trainees did not have experience in extracapsular cataract extraction (ECCE).

Cases were selected based on the following criteria: age 60–80, axial length 22.0–25.0 mm, endothelial cells per mm^2^ > 2000, anterior depth >2.5 mm, dilated pupil diameter >6 mm, cataract nuclear density graded between 2 and 4 (according to the Emery–Little classification [8]), and the absence of other oculopathies. Based on surgical experience, these cases were divided into two groups: the first 100 cases operated upon by each surgeon (this group included 24 cases of PCR) and the last 100 cases operated upon by each surgeon (this group included 15 cases of PCR).

### 2.2. Surgical Technique

All surgeries were performed under topical anesthesia. A standard 2.75 mm clear corneal incision was made at 11 o'clock, and a side port was created with a stab knife approximately 4 clock hours away. A continuous curvilinear capsulorhexis was performed, followed by hydrodissection and hydrodelineation. The Centurion Vision System (Alcon Laboratories Inc.) was used for ultrasonic emulsification. The phaco chop technique was employed to emulsify the nucleus (torsional power: 0–100%, vacuum, 380 mmHg, and aspiration flow: 35 cc/min). IA was then used to clear the cortex (vacuum: 500 mm Hg and aspiration flow: 35 cc/min). Sodium hyaluronate was injected into the anterior chamber, and a foldable IOL was inserted into the capsular bag. Finally, sodium hyaluronate was cleared by IA, and the corneal incisions were closed.

All operations performed by surgery trainees were supervised by an experienced phacoemulsification surgeon. When PCR occurred during surgery, the supervising surgeon took over and completed the operation. In instances when continuous curvilinear capsulorhexis was not successful, the supervising surgeon took over, and the case was excluded from analysis.

### 2.3. Data Collection

For each PCR case, the following clinical data were collected: the phase in which PCR occurred (the phaco phase or IA phase); the occurrence of vitreous loss, retinal detachment, and dropped nucleus; the site of IOL implantation; and postoperative best corrected visual acuity (BCVA, measured one week after surgery).

### 2.4. Statistical Analysis

Data were analyzed using SPSS software (version 13.0, International Business Machines Corp.). Fisher's exact test was used to evaluate differences in categorical variables, and Ridit analysis was used for the comparison of ranked datasets. The threshold of significance was set to a *P* value of 0.05.

## 3. Results

In the 1200 surgeries, there were 39 cases of PCR (3.25%). The occurrence rate of PCR was higher in the first 100 cases group (27 cases, 4.5%) than the last 100 cases group (12 cases, 2.0%) (*P*=0.015). PCR was more likely to occur in the phaco phase in the first 100 cases group and in the IA phase in the last 100 cases group (*P*=0.012). Vitreous loss was also more frequent in the first 100 cases group (23 cases) than the last 100 cases group (5 cases) (*P*=0.017). There was no significant difference between the two groups in the occurrence rates of retinal detachment or dropped nucleus (*P* > 0.05) (Table 1).

Among the 39 PCR cases, 15 had the IOL implanted in the capsular bag, 23 had the IOL implanted in the ciliary sulcus, and one had the IOL sutured at the ciliary sulcus. There was no difference between the two groups in terms of the position of IOL implantation (*P* > 0.05) (Table 2).

There was also no difference in postoperative BCVA between the two groups (*P* > 0.05) Table 3.

## 4. Discussion

Phacoemulsification is currently the most common technique used to treat cataracts worldwide. PCR is a severe and frequent complication in this operation. Without suitable treatment, patients who experience PCR can suffer a loss in visual acuity as well as a range of additional complications.

In previous studies, the occurrence rate of PCR in phacoemulsification surgery has been calculated as 1.9–5.2% [3–5, 9]. Studies focusing on surgery trainees have found the occurrence rate to be at the higher end of this range: between 3.8% and 5.1% [10–14]. However, the PCR occurrence rate in our study was much lower (3.25%). We propose that this is due to three main reasons. First, all the cases selected for each surgery trainee were simple cataract cases, with an Emery–Little grade of nuclear density between 2 and 4. Previous studies have identified various risk factors for PCR, including advanced age (>80), small pupil size (≤3 mm), mature-brunescent cataracts, anterior chamber depth <2.5 mm, posterior polar cataracts, diabetic retinopathy, and a surgeon with less experience [15]. As surgeries were performed by trainees in our study, we excluded other risk factors for PCR. Similarly, Woodfield et al. demonstrated that residency year did not significantly influence intraoperative complication rates when controlling for case difficulty [16]. Second, the ultrasonic emulsification system used in this study was safer and more advanced, and the parameters set were relatively conservative. Although such settings can result in low efficiency, a conservative approach does increase the safety of surgery. Finally, all operations performed by surgery trainees were supervised by an experienced phacoemulsification surgeon. In most cases, the supervising surgeon would draw the trainee's attention to any intraoperative risks or dangers as soon as possible. Also, with supervision, surgery trainees would be expected to be more confident and relaxed during the operation.

In our study, the first 200 cases operated upon by each surgery trainee were divided equally into two groups. We found that the PCR occurrence rate was significantly lower in the last 100 cases group than in the first 100 cases group (*P*=0.015). Similar findings were observed in a previous study conducted by Yulan et al.: the occurrence rate of PCR was significantly lower when the ophthalmology residents performing the operations had more surgical experience [17]. We also found that most instances of PCR occurred in the phaco phase in the first 100 cases group, whereas most occurred in the IA phase in the last 100 cases group (*P*=0.012). This finding is in accordance with our understanding of the trainees' learning curve. For trainee surgeons, nucleus emulsification is initially a difficult stage to master, and the release of energy at an inappropriate time or location in this stage is the main cause of PCR among less-experienced surgeons. After trainees master the principle of nucleus emulsification, the likelihood of complications in this step declines. Therefore, in the last 100 cases group, PCR was more frequently observed in the IA phase. Similarly, for more-experienced surgeons, PCR is more likely to occur in the IA phase than in the phaco phase.

In the 39 PCR cases, only one IOL was sutured at the ciliary sulcus. In this case, the decision to suture was made because the anterior capsule was miscut in the anterior vitrectomy. In all other cases, the IOL was implanted in either the capsular bag (in 15 eyes) or the ciliary sulcus (in 23 eyes). There was no difference between the two groups in terms of the site of IOL implantation. There was also no difference in BCVA between the two groups, though this may be due to the fact that the operations were taken over by an experienced surgeon after the occurrence of PCR.

In our study, all surgery trainees had no prior experience of ECCE. This is consistent with findings reported by Briszi et al. [14]. Due to the relative merits of phacoemulsification, almost all of the cataract patients in the hospital where the trainees worked underwent phacoemulsification surgery instead of ECCE. Consequently, the surgery trainees had received no opportunity to learn the technique of ECCE.

After mastering the processes of incision, capsulorhexis, IA, and IOL implantation, the surgery trainees were able to complete phacoemulsification surgery under the supervision of an experienced surgeon. In the hospital in which this study was conducted, surgery trainees are able to complete the entire operation without the supervision of an experienced surgeon after 200 completed cases. Similarly, the Royal College of Ophthalmologists requires trainees to complete 350 of these operations before a certificate of completion of training can be issued [18]. However, it is important to note that in the event of PCR, the supervising surgeon usually takes over from the surgery trainees. Therefore, the trainees may have few opportunities to manage PCR themselves. This situation was reflected in our study: even after 200 completed cases, surgery trainees cannot be confident in managing PCR.

## 5. Conclusions

In summary, as long as trainees are allocated with appropriate cataract cases and can work under the supervision of an experienced phacoemulsification surgeon, they can safely complete phacoemulsification surgery with a reasonably low occurrence rate of PCR. In this study, the level of experience of the trainee surgeons affected the phase in which PCR typically occurred as well as the likelihood of vitreous loss. It is a lengthy process for surgery trainees to reach the stage at which they can manage PCR and complex cataract surgery independently.

## Figures and Tables

**Table 1:** PCR in surgery.

PCR		Total	First 100 cases	Last 100 cases	*P* value
Cases (%)		39 (3.25%)	27 (4.5%)	12 (2.0%)	0.015
Phase	Phaco	25	21	4	0.012
	IA	14	6	8	
Vitreous loss	Yes	28	23	5	0.017
	No	11	4	7	
Retinal detachment	Yes	2	2	0	>0.05
	No	37	25	12	
Dropped nucleus	Yes	1	1	0	>0.05
	No	38	26	12	

PCR = posterior capsule rupture; IA = irrigation/aspiration.

**Table 2:** Situation of IOL implanting.

		Total	First 100 cases	Last 100 cases	*P* value
Situation of IOL implanting	Capsular bag	15 (38.4%)	8	7	>0.05
	Ciliary sulcus	23 (59.0%)	18	5	
	Sutured	1 (2.6%)	1	0	

**Table 3:** Postoperative visual acuity.

		Total	First 100 cases	Last 100 cases	*P* value
BCVA	<0.1	2 (5.1%)	2	0	>0.05
	0.1–0.5	14 (35.9%)	10	4	
	>0.5	23 (59.0%)	15	8	

BCVA = best corrected visual acuity.

## Data Availability

The data used to support the findings of this study are included within the article.
